# Transanal endoscopic microsurgery for rectal lesions in a specialist regional early rectal cancer centre: the Mersey experience

**DOI:** 10.1111/codi.14730

**Published:** 2019-07-01

**Authors:** M. Ondhia, P. Tamvakeras, P. O'Toole, A. Montazerri, T. Andrews, C. Farrell, S. Ahmed, S. Slawik, S. Ahmed, the Merseyside Early Rectal Cancer Network

**Affiliations:** ^1^ Royal Liverpool and Broadgreen University Hospital NHS Trust Liverpool UK; ^2^ Aintree University Hospital NHS Foundation Trust Liverpool UK; ^3^ Clatterbridge Cancer Centre NHS Foundation Trust Wirral UK

**Keywords:** Early rectal cancer, local excision rectal cancer

## Abstract

**Aim:**

Organ‐preserving local excision by transanal endoscopic microsurgery (TEM) for early rectal cancer offers significantly lower morbidity as compared to formal rectal cancer resection with acceptable outcomes. This study presents our 6‐year experience of TEM for rectal lesions referred to a specialist early rectal cancer centre in the UK.

**Method:**

Data were collected for all patients referred for TEM of suspected early rectal cancer to a regional specialist early rectal cancer multidisciplinary team (MDT) over a 6‐year period.

**Results:**

One hundred and forty‐one patients who underwent full‐thickness TEM for suspected or confirmed early rectal cancer were included. Thirty patients were referred for TEM following incomplete endoscopic polypectomy. Final pathology was benign in 77 (54.6%) cases and malignant in 64 (45.4%). Of the 61 confirmed adenocarcinomas, TEM resections were pT0 in 17 (27.9%), pT1 in 32 (51.7%), pT2 in 11 (18.0%) and pT3 in 1 (1.6%). Thirty‐eight of 61 patients (62.3%) had one or more poor histological prognostic features and these patients were offered further treatment. Twenty‐three of 61 (37.7%) patients with rectal adenocarcinoma required no further treatment following TEM. Forty‐three cases of rectal adenocarcinoma were available for establishing recurrence rates. Two of 43 patients (4.7%) developed a recurrence at a median follow‐up of 28.7 months (12.1–66.5 months). The overall estimated 5‐year overall survival rate was 87.9% and the disease‐free survival rate was 82.9%.

**Conclusion:**

Acceptable outcomes are possible for TEM surgery with appropriate patient selection, effective technique, expert histopathology, appropriate referral for adjuvant treatment and meticulous follow‐up. This can be achieved through an early rectal cancer MDT in a dedicated specialist regional centre.

**What does this paper add to the literature?**

This paper demonstrates that implementation of an early rectal cancer multidisciplinary team in a dedicated specialist centre allows transanal endoscopic microsurgery to be utilized as an appropriate method of treatment for early rectal cancer, with a low complication rate and acceptable oncological outcomes.

## Introduction

Surgical approaches for early rectal cancer have mainly focused on radical oncological resection by total mesorectal excision (TME) 1. Over the past two decades, local excision of early rectal cancer has become an accepted treatment in selected patients, with the advantages of reduced postoperative morbidity and mortality, less impairment of quality of life and equivalent oncological results compared with radical surgery 2, 3, 4, 5. Importantly, there is evidence that recurrence and survival rates after local excision are comparable with those of radical surgery 6, 7. Transanal endoscopic microsurgery (TEM) allows full‐thickness local excision of rectal lesions up to 20 cm from the anal verge under direct magnified vision with sufficient margins of surrounding normal healthy tissue 8. TEM is recommended for early rectal cancer with good oncological criteria: confined to the submucosa, well to moderately differentiated, no lymph node invasion, no lymphovascular invasion, 4–15 cm from the dentate line 9, 10.

Correct selection of patients for TEM is vital and multidisciplinary input is important to achieve this. Only early rectal cancers should be treated by local excision, and depth of infiltration (T stage), risk of lymph node involvement (N stage) and histological features (grade of differentiation and lymphovascular invasion) of the tumour, together with patient characteristics, inform the decision to consider the use of TEM. The aim of this study was to report the experience in our regional network of the use of TEM for the management of benign and malignant rectal neoplasms.

## Method

**Figure 1:**
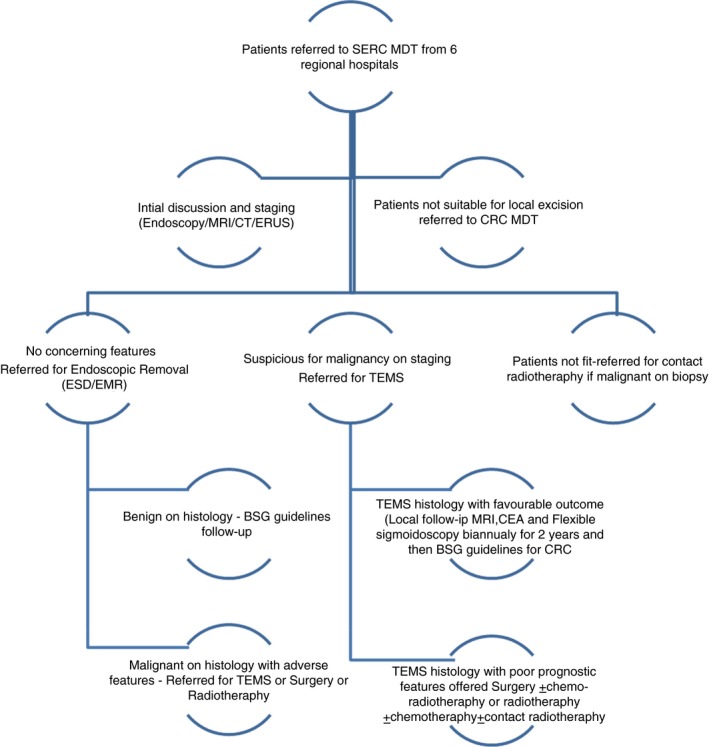
Flow chart demonstrating the working of the SERC MDT.

In 2011 a specialist early rectal cancer (SERC) multidisciplinary team (MDT) was set up serving the population of Merseyside and Cheshire, approximately 2.5 million people. The cancer network encourages referral of all patients with Stage 1 rectal cancer in line with National Institute for Health and Care Excellence recommendations 11. Large or complex rectal polyps where there is a risk of cancer are also referred. The SERC MDT comprises colorectal surgeons and endoscopists with a special interest in local excision techniques, oncologists, radiologists, pathologists and colorectal nurse specialists. Suitability for local excision was discussed for each case, and on average every patient was discussed at least three times before local excision. In each case, magnetic resonance imaging (MRI) and endorectal ultrasound (EUS) were used to confirm the tumour stage and identify local nodal disease. MRI was undertaken in all but two cases; one patient had claustrophobia and another had a pacemaker. EUS was undertaken by two SERC‐dedicated gastroenterologists. All patients underwent EUS unless they had undergone previous polypectomy/endoscopic mucosal resection (EMR) prior to referral to the SERC MDT. This policy was due to the expectation of limited views secondary to postprocedural scarring and fibrosis. All patients underwent computed tomography (CT) of the chest, thorax and abdomen to identify distant metastases. All patients were further assessed with flexible sigmoidoscopy by the core members of the SERC MDT. A regional SERC MDT referral proforma was developed that included endoscopic reports with colour images, biopsy reports, radiological imaging and patient history. The final decision on selection of cases for local excision was dependent on a combination of endoscopic assessments, biopsy reports, EUS, CT and MRI performed or reviewed by the core members. All cases that were referred for TEM by the SERC MDT were performed within 4 weeks of referral. Figure 1 shows a flow chart of the working of the SERC MDT.

This study included patients referred to the regional SERC MDT who underwent TEM for a rectal lesion from January 2011 to December 2016. A total of 14 patients who received neoadjuvant therapy prior to TEM, five of whom were part of the TEM and radiotherapy in early rectal cancer (TREC) trial, were excluded. Some patients with putatively benign adenomas were offered TEM based on endoscopic, radiology and EUS findings. For confirmed cancers, TEM was offered only for those patients with clinical T1 or T2 node‐negative disease.

Prior to the procedure patients were counselled in detail about the benefits and risks involved and written informed consent was obtained. All procedures were undertaken by two consultant colorectal surgeons (SA and SS) experienced in the use of either the Transanal Endoscopic Microsurgery (Richard Wolf Company, Tubingen, Germany) or Transanal Endoscopic Operation system (Karl Storz GmbH, Tuttlingen, Germany). In our series, all procedures were performed under general anaesthesia. The SERC MDT felt that this is safer practice and we don't have experience of performing TEM under spinal anaesthesia. However, the MDT agreed that most procedures could be performed under spinal anaesthesia by an experienced team. The procedure was carried out as described by Buess 12 and full‐thickness excisions were performed in all cases. Patients with proven cancer on preoperative histology staging but who were not fit for surgery or who refused surgery were referred for contact radiotherapy and were not included in the analysis.

Patients with adverse outcomes following TEM were offered either salvage surgery with or without chemoradiotherapy or radiotherapy with or without chemotherapy. Patients were counselled by a core member of the MDT at two separate outpatient appointments. Patients were informed about the merits and disadvantages of both treatment modalities. Due to postoperative changes following full‐thickness excision, a 6–12‐week interval was observed prior to salvage surgery if required.

Resected specimens were examined by dedicated consultant pathologists. Tumour staging was performed using the TNM classification. The Kikuchi level of invasion, cell differentiation, evidence of lymphovascular invasion and margin status were reported. At our institution, the SERC pathologists used the Kikuchi staging classification with the Royal College of Pathologist (RCPath) dataset 13 which is widely used in UK practice. Further treatment, radical surgery, chemotherapy, radiotherapy or a combination of these modalities, was offered to all patients with one or more adverse pathological features, namely tumour invasion beyond the submucosal layer, lymphovascular invasion, poorly differentiated tumour cells or positive resection margins (R1).

Follow‐up was in accordance with our institutional guidelines. Clinical follow‐up was at 6 weeks for all patients. Patients with a histologically confirmed benign lesion underwent biannual sigmoidoscopy for 2 years. In cases of malignant lesions, patients were followed up with flexible sigmoidoscopy every 4–6 months for 2 years, with MRI of the rectum twice a year for 2 years and measurement of serum carcinoembryonic antigen twice a year. All patients in this study were followed up for a minimum of 12 months. Local recurrence was defined as the development of a tumour at or near to the site of TEM more than 6 months following TEM and confirmed by biopsy. If a patient died the cause of death was obtained from hospital records and defined as either related or unrelated to rectal cancer.

Discrete variables are expressed as counts and percentages. Continuous variables are shown as mean ± standard deviation or median (range). Kaplan–Meier curves were calculated to determine disease‐free and overall survival. Statistical analyses were performed using the Statistical Package for the Social Sciences (SPSS) 24.0 for Windows (SPSS, Chicago, Illinois, USA).

## Results

Over a 6‐year period, 155 rectal lesions were excised by TEM. A total of 14 patients who received neoadjuvant therapy for downstaging of an advanced tumour prior to TEM were excluded from the analysis. Of the remaining 141 patients, 30 were referred for TEM following previous attempts at endoscopic resection. Table 1 summarizes patient demographics and lesion characteristics of all patients. The median age of our patients was 70 years (range 27–88 years) and 101 (71.6%) were men. Median American Society of Anesthesiologists status was 2 (range 1–3) and body mass index was 27.4 kg/m^2^ (range 16.6–37.9 kg/m^2^). The median distance from the anal verge was 6 cm (range 1–12 cm). Twenty‐six lesions (18.4%) were low (< 4 cm), 72 (51.1%) were mid‐rectal (4–8 cm) and 43 (30.5%) were in the proximal rectum (> 8 cm). The position of the rectal lesion was anterior in 36 (25.2%) cases, posterior in 60 (42.6%), lateral in 41 (29.1%) and circumferential in 4 (2.8%). The median lesion diameter was 35 mm (range 3–80 mm). Preoperative biopsy suggested a benign adenoma with low‐grade dysplasia in 48 (36.9%) cases, benign adenoma with high‐grade dysplasia in 41 (31.9%), adenocarcinoma in 44 (31.2%) and neuroendocrine tumour in 3 (2.1%). Table 2 demonstrates the correlation between preoperative imaging and pathological staging.

**Table 1:** Patient demographics and lesion characteristics.

Variable	Data (*n* = 141)
Patient characteristics	
Age (years), median (range)	70 (20–88)
Sex, *n* (%)	
Male	101 (71.6%)
Female	40 (28.4%)
Body mass index (kg/m^2^), median (range)	27.4 (16.6–37.9)
American Society of Anesthesiologists status, median (range)	2 (1–3)
Lesion characteristics	
Distance from anal verge (cm), median (range)	6 (1–12)
Location, *n* (%)	
Low rectum, < 4 cm	26 (18.4%)
Mid‐rectum, 4–8 cm	72 (51.1%)
Proximal rectum, > 8 cm	43 (30.5%)
Lesion diameter (mm), median (range)	25 (3–80)
Position of lesion, *n* (%)	
Anterior	36 (25.5%)
Posterior	60 (42.6%)
Lateral	41 (29.1%)
Circumferential	4 (2.8%)
Preoperative biopsy, *n* (%)	
Benign adenoma, low‐grade dysplasia	48 (36.9%)
Benign adenoma, high‐grade dysplasia	41 (31.9%)
Adenocarcinoma	44 (31.2%)
Neuroendocrine tumour	3 (2.1%)
Not available	5 (3.5%)

**Table 2:** Correlation between imaging and pathological staging.

	Imaging staging			
	uT0	uT1	uT2	uT3
Pathological staging				
pT0	2	11	4	0
pT1	2	16	13	1
pT2	0	3	7	1
pT3	0	0	1	0

### Postoperative results

There was no 90‐day mortality and 12/141 (8.5%) patients had postoperative complications. Delayed bleeding in the postoperative period occurred in five patients, of whom two required blood transfusions and the remaining three were managed conservatively. Pelvic sepsis occurred in one patient and was managed with an extended course of antibiotics. One patient had postoperative pyrexia with no source of infection and was managed conservatively. Five patients developed urinary retention. No patients developed anal incontinence or stenosis. The median length of hospital stay was 3.5 days (range 1–11 days).

### Pathology results

Overall, histological examination of the TEM specimen demonstrated an adenoma with low‐grade dysplasia in 41 cases, high‐grade dysplasia in 36, adenocarcinoma in 61 and neuroendocrine tumour in 3. Table 3 summarizes the postoperative histopathological staging of TEM specimens.

**Table 3:** Histology of TEM specimen (*n* = 141).

Postoperative histology	*n* (%)
Benign adenoma, low‐grade dysplasia	41 (29.1%)
High‐grade dysplasia	36 (25.5%)
Neuroendocrine tumour	3 (2.1%)
Adenocarcinoma	
pT0	17 (12.1%)
pT1	
sm1	3 (2.1%)
sm2	12 (9.9%)
sm3	17 (12.1%)
pT2	11 (7.8%)
pT3	1 (0.7%)

All patients who had malignant histology either prior to or following TEM were analysed in the cancer group. A total of 41 patients with rectal adenocarcinoma underwent TEM with no prior intervention, of which final histology found 31 pT1 and 10 pT2 cancers. Twenty patients underwent TEM for lesions where endoscopic resection had shown malignant histology with uncertain histological margins. Post‐TEM histology in these patients found no residual malignancy (pT0) in 17 cases and adenocarcinoma in 3 (one pT1, one pT2 and one pT3). Overall, postoperative histopathological staging of adenocarcinomas was as follows: 17 pT0, 32 pT1, 11 pT2 and 1 pT3. The Kikuchi level of invasion in 32 pT1 tumours was sm1 in 3 cases, sm2 in 12 and sm3 in 17. All three neuroendocrine tumours were stage pT1, excised completely and required no further surgical intervention.

Negative resection margins were demonstrated in 129/141 (91.5%) cases. Of 12 cases with positive resection margins, five were observed in patients with an adenocarcinoma (three pT1, two pT2 and one pT3), giving a positive resection margin rate of 5/61 (8.2%). All went on to have radical surgery and no cancer recurrences were observed in this group. Seven patients who had a benign adenoma with positive resection margins were closely monitored and no recurrences were observed during the study period.

### Salvage therapy following TEM

The local excision specimen showed one or more poor prognostic features in 38/64 (59.4%) patients: invasion into the deep third of the submucosal layer (Sm3) was identified in 29 cases, lymphovascular invasion in nine, positive resection margins in five and poor differentiation in one.

Of the patients with poor prognostic features, 38 (92%) received further treatment and three patients with adverse histology declined further treatment. Immediate salvage surgery was undertaken in 13 patients, by means of anterior resection in eight patients and abdominoperineal excision (APR) in five. Two of the 13 (both with pT0 disease on TEM) had suspicions of regional lymph node metastasis on imaging following TEM and received neoadjuvant radiotherapy prior to salvage surgery. Postoperative tumour staging following salvage surgery found pT0 disease in 11 cases and pT1 sm3 in 2. Five of 13 patients were found to have lymph node‐positive disease and all received adjuvant chemotherapy. Table 4 outlines the TEM specimen characteristics and outcomes for 13 patients who had salvage surgery. A total of 22 patients with adverse histology received either long‐course chemoradiotherapy (*n* = 9) or contact radiotherapy (*n* = 13) following TEM.

**Table 4:** Characteristics and pathological outcomes for patients who underwent salvage surgery after TEM (*n* = 13).

Patient	Postoperative stage	Resection margin	Lymphovascular invasion	Salvage therapy	Pathological stage	
					Tumour	Lymph node
1	pT1 sm3	Negative	Positive	APR	pT0	pN0 (0/10)
2	pT1 sm1	Negative	Positive	LAR	pT0	pN0 (0/11)
3	pT2	Positive	Negative	LAR	pT0	pN0 (0/12)
4	pT1 sm3	Positive	Positive	AR	pT0	pN0 (0/14)
5	pT0	Negative	Negative	NART + APR	ypT0	ypN0 (0/15)
6	pT2	Negative	Negative	APR	pT0	pN0 (0/9)
7	pT1 sm2	Positive	Negative	AR +ACT	pT0	pN1 (1/12)
8	pT3	Positive	Negative	AR + ACT	pT0	pN1 (1/14)
9	pT0	Negative	Negative	NART + AR + ACT	ypT0	ypN1 (1/26)
10	pT1 sm3	Negative	Positive	LAR + ACT	pT0	pN1 (2/17, apical node negative)
11	pT2	Positive	Negative	LAR + ACT	pT0	pN1 (3/22, apical node negative)
12	pT1 sm3	Negative	Negative	APR	pT1 SM3	pN0 (0/20)
13	pT1 sm3	Negative	Positive	APR	pT1 SM3	pN0 (0/26)

pT0, no evidence of primary tumour; pT1, tumour invades the submucosa; pT2, tumour invades the muscularis propria; pT3, tumour invades through the muscularis propria into the subserosa; sm1, tumour invades the superficial third of the submucosa; sm2, tumour invades the middle third of the submucosa; sm3, tumour invades the deep third of the submucosa; N0, no regional lymph node metastasis, N1, metastasis in one to three regional lymph nodes; AR, anterior resection, LAR, low anterior resection; APR, abdominoperineal excision; NART, neoadjuvant radiotherapy; ACT, adjuvant chemotherapy.

### Oncological outcomes of TEM

Of 61 patients with confirmed adenocarcinoma, 18 (29.5%) were excluded from further analysis: 13 patients who underwent immediate salvage surgery, three patients who refused further treatment despite adverse features on TEM histology, one patient who had TEM for a palliative indication and one patient who developed rectal cancer away from the TEM site and a synchronous left colon cancer. Forty‐three patients who had TEM with curative intent for a rectal adenocarcinoma were available for recurrence and mortality analysis.

**Figure 2:**
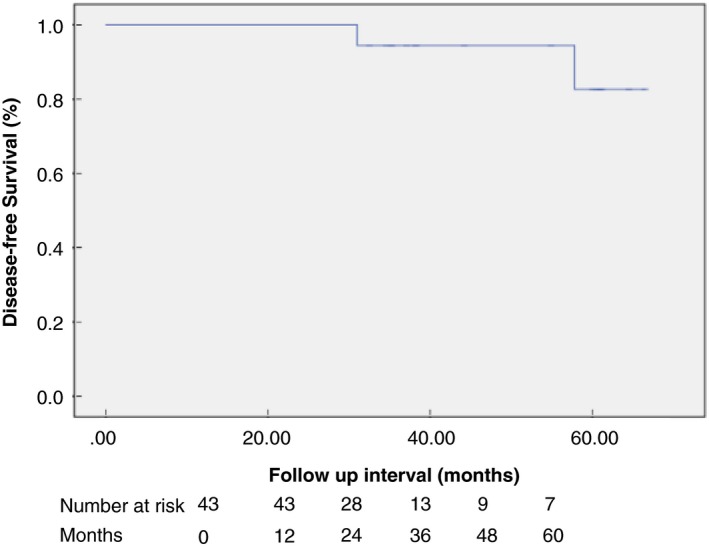
Kaplan–Meier estimation of disease‐free survival.

**Figure 3:**
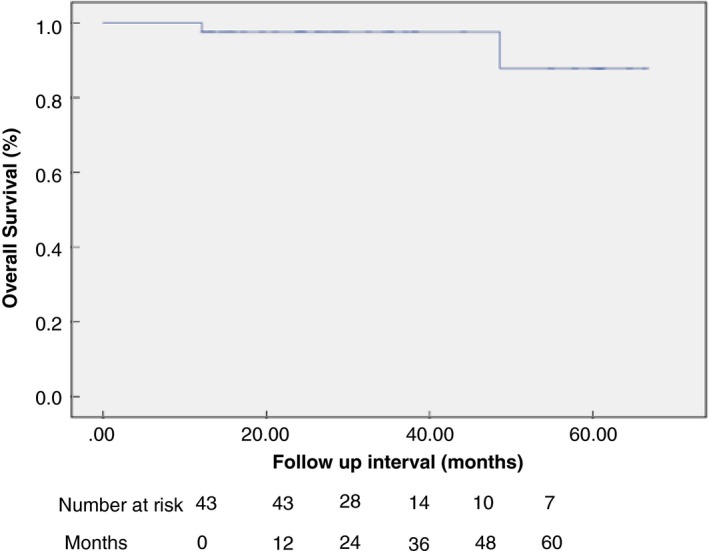
Kaplan–Meier estimation of overall survival.

Accounting for exclusions, there were two local recurrences and no metastatic recurrences at a median follow‐up of 28.7 months (range 12.1–66.5 months). Therefore, the overall recurrence rate was 2/43 (4.7%). In both cases of recurrence, TEM histology was pT1 sm2 with no adverse histological features. The first patient developed a recurrence at 17 months; he received neoadjuvant chemoradiotherapy then underwent APR, which found no residual malignancy. The second patient developed a recurrence at 31 months; he underwent long‐course chemoradiotherapy with complete response and had no surgical intervention. By Kaplan–Meier analysis, the disease‐free survival rate 5 years post‐TEM was 82.9%, as shown in Fig. 2. Three patients who were found to have pT2 disease on the TEM specimen refused any additional treatments (surgery or radiotherapy) and all developed recurrence in a median follow‐up of 18.5 months. Without exclusions, there was one metastatic and five local recurrences during the follow‐up period among 61 patients, giving a recurrence rate of 6/61 (9.8%.) two patients who had TEM of rectal cancer died during the follow‐up period; one death was related to rectal cancer. By Kaplan–Meier analysis the overall survival rate 5 years post‐TEM was 87.9%, as shown in Fig. 3. Patients with adverse histology of the TEM specimen opted for adjuvant chemoradiotherapy or contact radiotherapy as opposed to further surgical intervention. These patients all underwent endoscopy and biopsy following completion of adjuvant therapy to assess remission. No malignant recurrences were observed in this group at median follow‐up of 26.8 months (range 12.3–61.2 months). Patients underwent salvage surgery following TEM; of these, five patients with node‐positive disease also received adjuvant chemotherapy. Two recurrences were observed in this group. One patient developed a local recurrence with liver metastases 24 months after salvage surgery. The TEM specimen in this patient was pT1 sm3, R0, LVI positive. Following TEM he underwent anterior resection which showed no residual disease and 2/17 positive nodes for which he received adjuvant chemotherapy and disease remission was confirmed with follow‐up imaging and endoscopy. Following development of recurrence he underwent APR and further chemotherapy. The second patient developed a metastatic recurrence to the lungs 55 months following salvage surgery. In this patient, TEM was undertaken as completion following incomplete polypectomy. The TEM specimen showed pT0 disease, R0 and LVI negative; however, a suspicious node was detected on follow‐up imaging and he underwent neoadjuvant radiotherapy followed by APR. The APR specimen was ypT0 with no positive nodes. He received chemotherapy for liver metastases. Both patients described were still alive at the time of writing.

Patients who underwent TEM following previous attempted endoscopic removal (*n* = 30) were included in the oncological outcomes analysis. Thirteen of these patients were found to have residual adenocarcinoma and 17 (pT0) had no residual tumour. Interestingly, two of these 17 patients developed nodal disease recurrence in the follow‐up and both underwent salvage TME surgery. There was no recurrence found in 13/30 patients who had TEM following endoscopic removal of polyps.

## Discussion

Case selection for TEM requires high‐quality radiological, endoscopic and pathological lesion assessment. The procedure itself should be only be carried out by operators with specific training and experience, and decisions about further treatment after local excision are complex. For these reasons, in our region all lesions being considered for TEM are referred to a specialist MDT. We wished to assess whether the MDT was achieving the desired short‐ and long‐term outcomes.

The significant benefit of TEM is reduced morbidity compared with radical surgery 14. In our series, most postoperative complications were short term and managed conservatively. Postoperative bleeding is a frequently reported complication of transanal endoscopic surgery, occurring in 0.5–4.1% of cases 15, 16, and we observed a similar rate (3.2%). Additional risks communicated to patients include anal incontinence, rectal stenosis and rectovaginal fistula and no patients in this study experienced these complications. Overall, our complication rate was 8.5% which is significantly lower than the 50% reported with radical surgery 16 and comparable with previous reports for TEM in the literature (5–21%) 17, 18, 19, 20, 21, 22.

Compared with traditional transanal excision techniques, TEM offers superior resection margins, less specimen fragmentation and superior oncological outcomes 23, 24. Incomplete resection margins are associated with residual tumour, recurrence and metastasis 25. In our series, complete resection margins were obtained in 91.5% of cases. All patients with adenocarcinoma and incomplete resection margins were followed up meticulously.

The median diameter of lesions in our series was 35 mm (range 3–80 mm) and 29/141 (21%) were less than 20 mm, therefore they may have been amenable to EMR/endoscopic mucosal dissection (ESD) 26. However, in our experience the majority of these lesions had seen previous attempts at EMR/polypectomy prior to referral to the MDT and they were not suitable for further EMR/ESD; therefore, discussion for TEM was preferred. In addition, some of the lesions < 20 mm demonstrated high‐grade dysplasia on biopsy, thus full‐thickness excision was decided upon. At inception of the SERC MDT our ability to offer ESD was limited; however, this has expanded quite considerably over the years and selected benign and malignant lesions have now been treated with ESD instead of TEM.

The risk of lymph node metastasis has been reported to range between 12% and 28% in T2 and between 36% and 79% in T3 disease 27. In accordance with published recommendations 9, all patients in our series with advanced lesions (pT2 and pT3), and pT1 disease with adverse features, were counselled on the risk of metastasis and recurrence following TEM and offered either formal resection or adjuvant therapy.

We found acceptable oncological outcomes for 43 patients who underwent TEM with curative intent and did not go on to have salvage surgery. The recurrence rate for pT1 disease in this series was 4.7% (2/43), which is comparable with other series reported in the literature (0–23%) 28, 29, 30. Both patients who developed a recurrence had pT1 sm2 and did not show adverse histological features in the TEM specimen. Further sub‐analysis of T2 tumours showed that 8 of 11 patients who underwent adjuvant additional therapy following TEMS had no recurrence. Three declined any additional treatment following TEMS and all had developed recurrence during the follow‐up period (median 18.5 months). The local recurrence rate for T2 tumours excised by TEMS is reported in the literature as 19–47% 31. Despite relatively very small numbers, these data support previous studies and emphasize the importance of further treatment following TEMS excision of pT2 disease 32, 33.

The management of advanced lesions and pT1 disease with adverse histology is controversial. When one or more adverse features are present the risk of lymph node invasion up to 20–30% 1. Many patients will be of advanced age with significant comorbidity, therefore, radical surgery may not be a viable option. In our study, 22 patients with at least one adverse histological feature underwent adjuvant therapy following TEM. There were no local or metastatic recurrences in this group at a median follow‐up of 29 months (range 12.1–61.2 months). Although these numbers are small and the follow‐up period relatively short, our findings are in line with previous studies which support the role of adjuvant therapy following TEMS 31. However, the existing literature is limited to small series with wide variation of recurrence rates (6–43%) 33, 34. The ongoing TESAR trial 34 will assess oncological outcome in patients receiving adjuvant therapy after TEMS compared with patients undergoing radical surgery.

In one case of TEM, final histology very unexpectedly demonstrated a pT3 tumour. The patient was referred from a regional hospital and had multiple EMR attempts over 2 years for what was presumed to be a benign lesion. All previous biopsies were benign low‐grade dysplasia with no focal high grade. The patient was referred for TEM to treat further recurrence of the polyp. Due to fibrosis and rectal stenosis secondary to previous EMR, MRI and EUS views were limited and suggested T2 and T1 disease, respectively, with no suspicious nodes. Final histology of the TEM specimen was pT3R1 and the patient underwent anterior resection followed by adjuvant chemotherapy for positive nodal disease. No recurrence was reported during a follow up of 38.5 months.

In our recurrence and survival analysis we have chosen to include patients with polypectomy specimen positive for malignancy but subsequently found to have no residual malignancy, i.e. pT0 disease. It could be argued that patients with negative residual tumour in the TEM specimen following polypectomy may represent successful management of the tumour lesion by endoscopic means only; however, without further study by means of a randomized‐controlled trial it remains speculative whether no further treatment in these patients would have the same outcome without TEM. Given the limited sample size we were unable to make a comparison of patients who underwent TEM alone and polypectomy + TEM, and acknowledge that this presents a limitation of our study.

In our series 41 of the 141 (29.1%) patients demonstrated high‐grade dysplasia on biopsy prior to TEM, and 25 of these 41 (61.0%) were proven to have malignant T1 cancer on final histology. The SERC MDT felt that full histological staging was essential in those patients and that could only be possible with full‐thickness local excision, and offering wait and watch outside of a clinical trial was not appropriate.

To select which lesions are amenable for local excision it is important to select diagnostic tools that define tumour staging with acceptable sensitivity and specificity. Where feasible, lesions were assessed by both MRI and EUS. Where MRI and EUS staging conflicted the MDT trusted EUS, in combination with endoscopic assessment by a core member of the MDT, for T staging due to its known better sensitivity and specificity 35, 36, 37. For nodal disease, MRI was considered more reliable 38; however, if the pre‐TEM targeted biopsies were negative for malignancy then nonspecific nodes were not considered significant. In our series, there were only a few cases where EUS and MRI conflicted (*n* = 9).

A total of 50% of the patients referred to the SERC MDT were considered to be suspicious and referred for full‐thickness rectal excision. Polyps that were considered likely to be benign were referred for EMR or ESD as appropriate, and the procedures were performed by the core members. Lesions on or close to the dentate line are often considered challenging for full‐thickness excision. In our series, lesions at or close to the dentate line were treated in exactly the same way as other lesions above it. In three cases, where lesions were at the dentate line the operating surgeon did the initial part of the procedure using a Lone Star retractor developed for use in few‐centimetre spaces with a TEMS proctoscope. In cases where the polyp was sited at the dentate line and considered benign patients were referred for ESD/EMR under local anaesthetic.

TME post‐TEM is considered challenging due to disruption of the TME plane and postoperative inflammation. In patients who required TME rectal surgery following poor adverse outcome on TEM excision, surgery was delayed from 6 to 12 weeks in order to reduce postsurgical inflammation. Only three patients underwent TME surgery following TEM; therefore, our experience is limited; however, we did not find any significant difference in the quality of the TME specimen.

One of the limitations of our study is the lack of data on quality of life and bowel function following local excision with and without radiotherapy and on patients who chose to have surgery following adverse outcome of local excision. These issues will require additional data and are beyond the scope of this paper.

This study demonstrates that oncological outcomes of TEM without the need for further treatment (favourable pathology) are acceptable. However, we acknowledge there are a proportion of patients who require additional treatment following TEM due to unfavourable prognostic factors. We consider that centralization of the management of SERC and additional expertise in local staging and treatment will in future enable clinicians to predict patients who are likely to have an unfavourable outcome post‐TEM. Our results suggest that with careful selection a significant majority of patients can avoid rectal cancer resection with its associated co‐morbidity and mortality, and that this can be achieved with acceptable oncological outcomes. During the study period no patients referred from the SERC for ESD/EMR were found to have a malignancy. This emphasizes the benefits of centralization of the service for early rectal cancer.

This regional SERC MDT has demonstrated the successful implementation of the early rectal cancer MDT model. We have demonstrated that acceptable outcomes are possible for TEM with careful patient selection, effective techniques, expert histopathology, appropriate referral for adjuvant treatment and meticulous follow‐up.

## Author contributions

ShA conceived the idea for the study. MO, PT and ShA performed data collection. ShA and SS were involved in planning and supervised data collection. MO conducted data analysis. MO and ShA drafted the manuscript. SS, AM, POT, TA, SuA contributed to writing of the manuscript. All authors discussed the results and gave final approval of the manuscript.

## Conflicts of interest

There are no conflicts of interest to declare.

## Ethical approval

This is a retrospective study of a standard treatment for early rectal lesion. Following consultation with our research, development and information governance department we were advised ethical approval was not necessary.
